# Effectiveness of computer-mediated interventions for informal carers of people with dementia—a systematic review

**DOI:** 10.1017/S1041610214001045

**Published:** 2014-07-03

**Authors:** Vicky McKechnie, Chris Barker, Josh Stott

**Affiliations:** Research Department of Clinical, Educational and Health Psychology, University College London, London, UK

**Keywords:** Dementia, Alzheimer’s disease, social support, carers

## Abstract

**Background:**

Caring for a friend or relative with dementia can be burdensome and stressful, and puts carers at increased risk of physical and psychological problems. A number of psychosocial interventions, some delivered by computer, have been developed to support carers. This review evaluates the outcomes of computer-mediated interventions.

**Methods:**

PsychINFO, MEDLINE, and CINAHL Plus were searched for papers published between January 2000 and September 2012. Study quality was evaluated using a modified version of Downs and Black's (1998) checklist.

**Results:**

Fourteen empirical studies, evaluating a range of complex, multifaceted interventions, met inclusion criteria. The most commonly measured variables were carer burden/stress and depression. In general, higher quality studies found that interventions did have an effect on these variables. Two higher quality studies also found that anxiety was reduced following intervention. Most studies found that positive aspects of caring were increased through these interventions, as was carer self-efficacy. There were mixed results in relation to social support, and physical aspects of caring did not seem to be affected. Program impact measures indicated general acceptability of these interventions.

**Conclusions:**

The findings support the provision of computer-mediated interventions for carers of people with dementia. Future studies would benefit from design improvements, such as articulating clearly defined aims, having a control group, having adequate statistical power, and measuring a greater range of factors important to carers themselves.

## Introduction

Many of the 750,000 people in the United Kingdom with dementia are cared at home by a relative or friend (Alzheimer's Society, 2011). Carers of people with dementia (PwD) frequently report feelings of social isolation and inadequate social support (Stoltz *et al.*, 2004) and the risk of anxiety and depressive disorders is increased (Schulz and Martire, 2004). Furthermore, these negative outcomes are correlated with the level of cognitive impairment as well as behavioral disturbance in the person with dementia (Schoenmakers *et al.*, 2010). Contextual factors are also important in experiences of social isolation, with lower socioeconomic status and poorer formal support for the person with dementia related to greater social isolation (Robison *et al.*, 2009). Older carers’ physical health (Schulz and Martire, 2004) and mortality (Schulz and Beach, 1999) can also be affected, which can in turn compromise their ability to care for the person with dementia.

A range of interventions for carers of PwD exist to mitigate these problems (Sörensen *et al.*, 2002). The present review is concerned with psychosocial information and communication technology (ICT) interventions aimed at providing information and improving the carer's wellbeing and coping skills (e.g., psychoeducational interventions and support groups) rather than interventions aimed at reducing the objective amount of care provided by carers (e.g., interventions that improve the patient's competence in daily activities).

Uptake of traditional dementia services, both for PwD and carers, has been low; furthermore, carers of PwD show great interest in support services other than traditional face-to-face support groups, for example, telephone support, newsletters, and interventions delivered on computer (Colantonio *et al.*, 2001). Such nontraditional interventions can be similar in content to traditional interventions, but vary in their format. For example, face-to-face educational sessions can also be delivered on video or DVD (see, e.g., Gant *et al.*, 2007). Nontraditional media also open up a range of possibilities for carer support. For example, internet support groups can help individuals physically distanced from one another, 24 hours a day and seven days a week. This is particularly helpful for individuals who are physically isolated or experiencing rare or stigmatized conditions (White and Dorman, 2001). The current review will focus on one form of nontraditional intervention—computer-mediated psychosocial interventions.

### Previous reviews

There have been three previous reviews of this area. Powell *et al.* (2008) conducted a systematic review of networked technologies supporting informal carers of PwD. They identified 15 papers that described five different multifaceted interventions. They found that the interventions had inconsistent outcomes, but suggested that they had moderate effects on improving carer stress and depression. Treatment effects varied with carer characteristics such as ethnic group, formal support, and baseline burden. This review was published as a brief descriptive report, and its evaluation of studies was therefore limited. In addition, with the rapid development of the internet and other computer technologies, it now requires updating.

A more recent French review (Wu *et al.*, 2009) looked at studies of information and communication technology interventions supporting carers of PwD. Sixteen papers concerning nine intervention programs (internet and telephone interventions) were described, without a critical analysis or comparison of studies, and with limited conclusions drawn.

Godwin *et al.* (2013) conducted a systematic review with the aim of ascertaining the psychosocial effects of technology-driven interventions for informal carers of PwD. Eight papers were evaluated. This review only included RCTs and excluded interventions that only used CDs. All the studies reviewed showed positive outcomes, but there was considerable variability in the content and delivery of interventions, as well as inconsistency in measurement and variability of outcome.

### Aims of this review

Computer-mediated intervention for carers of PwD is a growing area and a wide range of interventions have been developed. In order for future interventions to be maximally effective, and to ensure that service providers are aware of the strengths and weaknesses of different interventions, it is important that the current evidence base be evaluated. The present review therefore aims to address the question: How effective are computer-mediated psychosocial interventions for informal carers of PwD?

## Method

### Inclusion and exclusion criteria

The inclusion criteria were:

1. The intervention must be computer mediated. This could include DVDs, CD-ROMs, the internet, or computer programs, but excludes interventions that exclusively use the telephone. The intervention does not need to exclusively be computer mediated, but that must be its main component.
2. Interventions must be psychosocial interventions, including therapy, professional, or peer support programs, educational or information programs.
3. The carers must be informal, that is, they must not be paid carers such as nursing home staff. However, the PwD can be resident either at home or in an institution.
4. Studies must include dementia as the diagnosis of the person being cared for, but do not have to be limited to dementia. All types and all stages of dementia were included.
5. Studies must use at least one quantitative measure to assess the outcome of the intervention. These measures do not need to be standardized, and can be questionnaires asking for users’ views of the effects of the intervention. Studies using only questionnaires that consider features of the intervention, such as its convenience, user-friendliness, or accessibility, were excluded.
6. Studies can be randomized-controlled trials, pre- and posttest studies with or without a control group, or posttest-only studies with or without a control group. Case studies and qualitative research were excluded.
7. Studies must be published in peer reviewed journal articles, in English, from January 2000 to September 2012. The lower limit was chosen because the review focuses on current technologies.

### Search strategy

Search terms were organized into four conceptual areas:

computer* OR DVD* OR internet* OR network* OR technolog* OR ICT OR online* OR CD-ROM*

AND

intervention* OR train* OR therap* OR support* OR treatment*

AND

carer* OR caring * OR caregiv* OR family* OR families* OR parent* OR mother* OR father*

AND

dement* OR Alzheimer*

**Figure 1.**
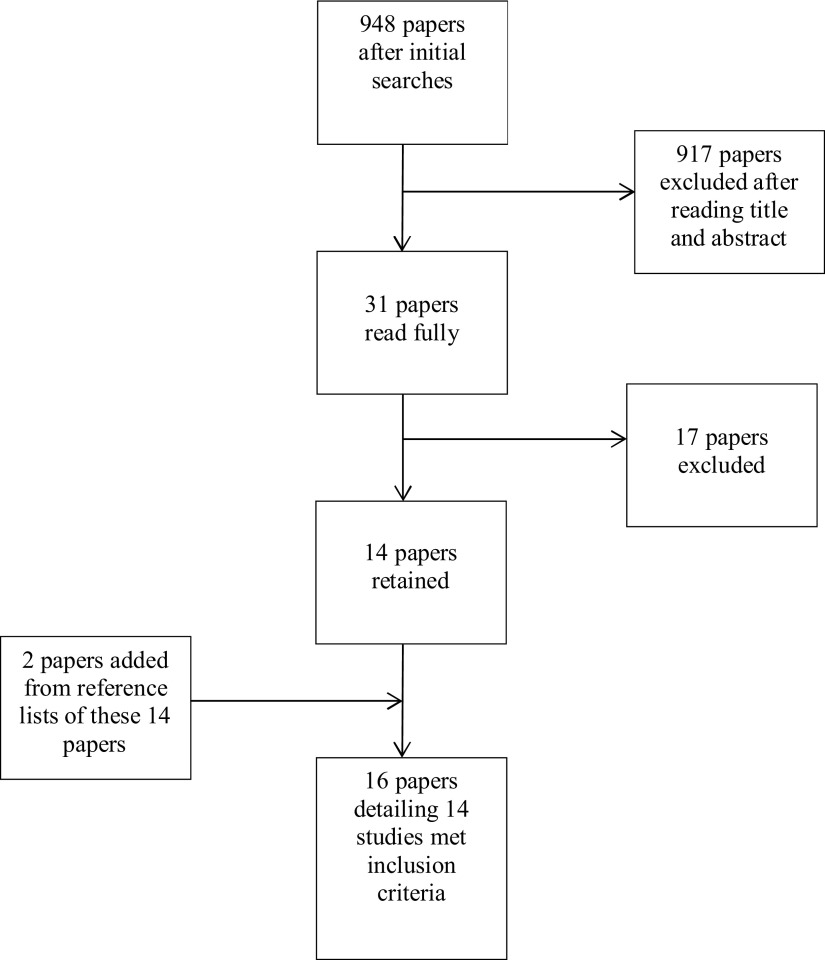
Flow diagram of studies included and excluded.

Grey literature was not searched. Searches of PsychINFO, MEDLINE, and CINAHL Plus databases generated 262, 708, and 248 papers, respectively. After de-duplication, there were 948 papers of which the titles and abstracts were examined, out of which 31 papers seemed that they might potentially fulfill the inclusion criteria; the full texts of these were then read in full by the first and third authors (see Figure 1 for PRISMA flowchart).

Seventeen of these 31 papers were excluded. Seven were excluded because the intervention was not computer mediated, four because the evaluation of the intervention was limited to anecdotal reports, qualitative interviews, and/or usage or intervention acceptability information, one was a literature review, one was a description of an intervention, one was a research proposal, one was a study of the relative importance of intervention elements such as social support, one was a meta-analysis, and one investigated whether older carers were able to complete standardized questionnaires. There were queries about four papers’ eligibility, and in these instances, the second author was consulted in order to reach consensus.

This left 14 papers for review. In two instances, two papers reported results from the same study (Glueckauf and Loomis, 2003; Glueckauf *et al.*, 2004; Chiu *et al.*, 2009; Chiu and Eysenbach, 2010). So, at this point, 12 studies were identified for review. For clarity, when these studies are referred to, Chiu *et al.* (2009) and Glueckauf *et al.* (2004) will be cited.

Reference lists from the included papers, as well as from Powell *et al.* (2008) and Wu *et al.* (2009), were examined to see whether any additional papers were eligible. This led to four further papers being examined, yielding two additional papers (Magnusson *et al.*, 2005; Marziali and Donahue, 2006). The reference lists of these papers were also searched, leading to one further paper being examined, but no further studies identified. Sixteen papers, representing 14 empirical studies were evaluated.

### Study quality

The quality of studies was evaluated using a modified version of Downs and Black's (1998) checklist for the assessment of the methodological quality both of randomized and nonrandomized studies of health care interventions. In light of the nature of the studies being evaluated, several items on the checklist were not relevant (items 8, 13, 14, and 19), and were therefore excluded from the assessment. For example, item 14 asked whether any attempt was made to blind study subjects to the interventions that they received, which is not relevant for most psychological intervention studies. Each study could achieve a score of between 0 and 24, with higher scores indicating better quality. Scores of 0–8 were considered to indicate studies of low quality; 9–16 as medium quality, and 17–24 as high quality.

## Results

Table 1 summarizes the 14 studies evaluated. Several used mixed methods; the present review only addresses their quantitative components.

**Table 1.** Study characteristics

authors and date	participants	initial sample size*	intervention	aims of study	measures	design	principal findings^	quality score^†^
Beauchamp *et al.* (2005)	Employed family carers in USA	329	Worksite-based internet multimedia program: “Caregiver's Friend: Dealing with Dementia.” Program provided text materials and videos that modeled positive caregiving strategies. 30 days exposure (Control: wait list)	Can exposure to the program: (a) improve carer appraisals?; (b) increase the use of constructive coping skills?; (c) reduce the negative outcomes of depression, anxiety and strain?; (d) increase positive perceptions of caregiving?	Items used from: CES-D; STAI; CSI; PAC; RWC	Pretest–posttest RCT; Follow up at end of intervention	Treatment group reported greater gains with respect to measures of self-efficacy, intention to get support and caregiver gain, and reductions in carer stress, strain, depressive symptomatology, and state anxiety	20
Chiu *et al.* (2009)	Chinese Canadian family carers of elderly people with Alzheimer's disease and related dementias	35	Internet-based Caregiver Support Service (ICSS): An online carer information handbook, and exchange of e-mails between caregivers and Chinese professional clinicians (occupational therapists) 6 months access	To explore usage behavior associated with outcomes and to evaluate effects of participation in ICSS on carer health outcomes	BSFC	Single group pretest–posttest Follow up at end of intervention	Nonusers had an increase in perceived burden postintervention, while frequent users had a decrease of burden score	12
Eisdorfer *et al.* (2003)	Cuban American and White American family carers of people with Alzheimer's Disease and related dementias	225	Part of the REACH (Resources for Enhancing Alzheimer's Caregiver Health) program. 3 conditions: (a) structural ecosystems therapy (b) structural ecosystems therapy + computer–telephone integration system (c) minimal support condition 12 month interventions.	To examine the efficacy of the two interventions across Cuban American and White American carers and to examine the efficacy of the interventions over time. To explore the differential effects of the treatment across varying carer–care recipient dyads	CES-D	Pretest–posttest RCT; measures at baseline, 6, 12, and 18 months	Carers in condition (b) experienced reduction in depressive symptoms at 6 months relative to other conditions. 18-month follow up indicated the intervention was particularly beneficial for Cuban American husband and daughter carers.	18
Finkel *et al.* (2007)	Family carers of PwD	46	Provision of information about dementia and community resources and strategies to enhance safety, communication, self-care, social support, and management of problem behaviors—delivered through the customized Computer–Telephone Integration System 6 month intervention (Control: information only)	Evaluate intervention effectiveness	CES-D; three questions assessing the primary domains of the RMBPC; modified version of CHHBS; a Received Social Support Scale from the ISSB	Pretest–posttest RCT; 6 months follow up	Carers in intervention condition reported a decrease in burden post intervention, and those who evidenced high depression at baseline experienced a decline in depression	13
Gallagher-Thompson *et al.* (2010)	Chinese American carers living in the San Francisco Bay area	70	CBT skill training program delivered on a DVD (plus workbook) 12–16 week intervention. (Control: general educational DVD program on dementia, plus written materials to supplement)	Hypothesis—that the skill training DVD treatment would be more effective than the educational DVD in reducing conditional bother, increasing positive affect, and reducing other negative symptoms of depression	CES-D; RMBPC; Program evaluation questionnaire	Pretest–posttest RCT; follow up 16 weeks after baseline	The two interventions did not differentially affect level of depressive symptoms; positive affect subscale score of CES-D increased more for those in skill DVD group. Reaction to problems decreased in skill DVD group. Both groups reported the intervention improved their confidence and skills in caring, with the skill DVD group generally reporting greater gains.	18
Glueckauf *et al.* (2004)	Carers of individuals with progressive dementia from Maryland, Florida (bar one)	40	Alzheimer's Caregiver Support Online (AlzOnline)—an internet- and telephone-based education and support network for carer of individuals with progressive dementia 16 week intervention on average	Initial program evaluation of AlzOnline's Positive Caregiving Classes. Objectives: To assess the impact of the program on the psychosocial functioning of carer participants, particularly their perceptions of self-efficacy, emotional growth, and burden from the caregiving experience	CSES; SRGS; CAI	Single group pretest–posttest	Participants reported pre- to postclass increases on all 3 CSES subscales, and concomitant decreases in subjective carer burden. There were substantial improvements in their perceptions of self-efficacy in performing routine caregiving duties and managing challenging care recipient behaviors, and their appraisals of the emotional caregiver burden from pre- to posttesting phase	10
Lewis *et al.* (2010)	Carers of people with dementia in USA	63	Internet-Based Savvy Caregiver (ISBC)—an internet-based psychoeducational program	To establish feasibility and acceptability of program	Follow-up questionnaire	Single group posttest	Participants found the program educational, convenient, useful, and interesting. They endorsed feeling more confident in caring skills and communication with their family.	9
Magnusson *et al.* (2005)	Family carers of older people in two municipalities in the west of Sweden. Carers: 16 stroke, 5 dementia, 5 diabetes, and various other	34	Swedish ACTION (Assisting family Carers using Telematics Interventions to meet Older persons’ Needs) project: An ICT service providing carers with information, education, and support Intervention ranged from 3 months to 1 year.	Program evaluation	Modified version of the PREP evaluation questionnaire	Single group posttest	The intervention had a moderately positive effect on the preparedness, rewards, and satisfaction of caring	5
Mahoney *et al.* (2003)	Family carers of people with Alzheimer's Disease in USA	100	Part of the REACH (Resources for Enhancing Alzheimer's Caregiver Health) program. Technology intervention—access to a computer-mediated automated interactive voice response (IVR) intervention 12 month intervention (Control: usual care)	To investigate whether this system could reduce stress associated with caring for a family member with Alzheimer's related disruptive behaviors.	RMBPC; STAI; CES-D	Pretest– posttest RCT; measures at baseline, 6, 12, and 18 months	Participants with lower mastery at baseline showed improvement in bother, anxiety and depression. Wives showed a reduction of the bothersome nature of caregiving	21
Marziali and Donahue (2006)	Family carers in Canada of older adults with neurodegenerative diseases (Alzheimer’s; stroke-related dementia; Parkinson’s)	66	“Caring For Others” intervention program. Intervention group received computers and access to a website with links to information, e-mail and threaded discussion, video-conferencing link (→ 10-session manual-guided psychosocial support group facilitated by a group therapist, followed by 12 additional online sessions facilitated by a group member) 22 week intervention (Control: no intervention)	To evaluate the effects of the intervention on carers	HSQ-12; CES-D; a measure requiring carers to endorse presence/absence of activities of daily living and instrumental ADLs performed for care recipient, rating degree of stress for each; RMBPC; MSPSS	Pretest– posttest RCT; measures at baseline and 6-month follow up	Intervention group experienced a decline in stress	12
Marziali and Garcia (2011)	Canadian carers (French and English speaking) from three cities	91	Two internet interventions: a text-based chat group (including access to a carer information handbook and six videos on managing caregiving tasks); and video conferencing psychotherapeutic support group intervention facilitated by a clinician, plus access to a carer information handbook 6-month intervention for chat group; 20-week intervention for support group.	To examine the impact on dementia carers’ experienced stress and health status	HSQ-12; CES-D; SMAF	Two group pretest–posttest, with follow up at 6 months	In contrast to the chat group, the video group showed greater improvement in mental health status	14
Rosen *et al.* (2003)	Family members (“primary decision makers”) of nursing home residents with dementia living in nursing homes around Pittsburgh, Pennsylvania, USA.	18	Computer-based education on dementia, agitation/aggression, and carer stress 45-min intervention	To enhance family participation in nursing home care	Knowledge questionnaire	Single group pretest–posttest	Knowledge of the key principles of dementia care improved	8
Torp *et al.* (2008)	Elderly (≥60 years old) spousal carers of people with diagnosis of dementia or stroke, in Norway, who were computer novices. Carers: 14 cerebral stroke, 5 dementia	19	Training for carers on how to use and collect information from the Internet. All computers were connected in an online discussion forum. Also videophone contact between participants. Call centre run by professionals who provided support on use of ICT, and advice and support regarding participants’ caring situation. 12-month intervention	To explore whether use of ICT by informal carers of frail elderly people living at home would enable them to gain more knowledge about chronic illness, caring, and coping, establish an informal support network, and reduce stress and related mental health problems	FFCS; Revised UCLA Loneliness Scale; RSS; GHQ-20; questions about ICT use and knowledge about chronic disease and caring	Single group pretest–posttest with measures at baseline and 12 months	Carers reported extensive use of the ICT service, more social contacts and increased support and less need for information about chronic illness and caring	11
Van der Roest *et al.* (2010)	Carers of community dwelling PwD in Amsterdam	29	DEMentia-specific dynamic interactive social chart (DEM-DISC)—a demand-oriented web-based social chart for dementia care that aims to provide users with customized answers about potentially relevant care and support services in their region. (Control: access to pre-existing information sources)	To evaluate the impact on the daily life of carers and the usefulness of the first prototype of DEM-DISC.	SSCQ; PMS; a questionnaire on knowledge about care and welfare services; USE questionnaire.	Pretest posttest with control group (not randomized); follow up 2 months after baseline	Following the intervention, carers in the intervention group reported a higher feeling of competence than carers in the control group	16

^All findings reported in this table were statistically significant at *p* < .05, with the exception of Lewis *et al.* (2010) and Magnusson *et al.* (2005), who reported only descriptive statistics, as well as Torp *et al.’s* (2008) measure of forum usage, and Gallagher-Thompson *et al.’s* (2010) program evaluation questionnaire, which were both evaluated using descriptive statistics.

*i.e. sample size at the start of the study, before any participants dropped out.

^†^The quality of each study was evaluated using a modified version of Downs and Black's (1998) checklist for the assessment of the methodological quality of both randomized and nonrandomized studies of healthcare interventions. Each study could achieve a score of between 0 and 24, with higher scores indicating better quality. Scores of 0–8 were considered to indicate studies of low quality; 9–16 as medium quality, and 17–24 as high quality.

BSFC = Burden Scale for Family Caregivers (Gräsel *et al.*, 2003); CAI = Caregiver Appraisal Inventory (Lawton *et al.*, 1989); CES-D = Center for Epidemiologic Studies-Depression scale (Radloff *et al.*, 1977); CHHBS = Caregiver Health and Health Behaviours Scale (Posner *et al.*, 1993); CSES = Caregiving Self-Efficacy Scale (Steffen *et al.*, 2002); CSI = Caregiver Strain Instrument (*Bass et al.*, 1998); FFCS = Family and Friendship Contacts Scale (Andersson, 1984); GHQ-20 = General Health Questionnaire 20 (Goldberg and Williams, 1991); HSQ-12 = Health Status Questionnaire 12 (Pettit *et al.*, 2001); ISSB = Inventory of Socially Supportive Behaviours (Barrera *et al.*, 1981); MSPSS = Multidimensional Scale of Perceived Social Support (Zimit *et al.*, 1988); PAC = Positive Aspects of Caregiving (Tarlow *et al.*, 2004); PMS = Pearlin Mastery Scale (Pearlin and Schooler, 1978); PREP evaluation questionnaire (Archbold *et al.*, 1995); Revised UCLA Loneliness Scale (Russel *et al.*, 1980); RMBPC = Revised Memory and Behaviour Problems Checklist (Teri *et al.*, 1992); RSS = Relative Stress Scale (Greene *et al.*, 1982); RWC = Revised Ways of Coping (Vitaliano *et al.*, 1985); SMAF = Functional Autonomy Measurement System (Hébert *et al.*, 2003); SRGS = Stress-related Growth Scale (Parke *et al.*, 1996);

SSCQ = Short Sense of Competence Questionnaire (Vernooij-Dassen *et al.*, 1999); STAI = State Trait Anxiety Inventory (Spielberger *et al.*, 1985).

### Interventions

The interventions were varied; many had multiple components. In nine studies, with quality scores ranging from 5 to 20, the intervention was either primarily or entirely delivered as an internet program or resource. One high quality study used a computer-mediated automated interactive voice response intervention; two, of high and medium quality, used a computer–telephone integration system; one of high quality used a DVD program; and one low quality study used computer-, but not internet-, based education. In eight studies, the main part of the intervention was education; three combined education with professional therapy or support; two combined education with more general support; one compared education and professional support with education and more general peer support.

Their aims tended to be to reduce carer distress and increase competence in caregiving. One aimed to enhance family participation in nursing home care (Rosen *et al.*, 2003). Five studies were labeled as pilot or feasibility studies (Rosen *et al.*, 2003; Marziali and Donahue, 2006; Marziali and Garcia, 2006; Torp *et al.*, 2008; van der Roest *et al.*, 2010) and some that were not described as pilots nonetheless involved the development of a new intervention (e.g., Glueckauf *et al.*, 2004; Chiu *et al.*, 2009).

Six papers described interventions that included the ongoing involvement of a professional, six described interventions that did not, with one paper comparing an intervention including a professional with one that did not. Magnusson *et al.* (2005) was unclear about the level of professional involvement. In only one study did it seem that the intervention was continued after it was evaluated (Torp *et al.*, 2008). This is surprising, given that a benefit of computer-mediated interventions, particularly internet interventions, is that they can be used on an ongoing basis. Individual interventions varied in terms of the amount and frequency of input over the time period that they were offered. In most cases, carers knew the diagnosis of the care recipient but studies were not excluded if they did not.

### Study designs

Six studies were randomized controlled trials, one was a controlled trial, four used a single group pretest posttest design, two used a single group posttest-only design, and one used a two group pretest posttest design. Initial sample sizes ranged from 18 (Rosen *et al.*, 2003) to 329 (Beauchamp *et al.*, 2005), attrition rates ranged from 0% (Rosen *et al.*, 2003; Marziali and Garcia, 2011) to 42% (Marziali and Donahue, 2006), although several studies did not report them. Although a number of papers compared the characteristics (such as age and baseline depression score) of those who dropped out with those who did not, only one presented an intent-to-treat analysis (Chiu *et al.*, 2009) and one interpolated 18 months follow-up scores where data were available (Eisdorfer *et al.*, 2003).

No study provided a power calculation, and several authors noted that their study was likely to be underpowered. Several small studies used mixed-methods designs (e.g., Magnusson *et al.*, 2005; Torp *et al.*, 2008). Reporting of follow-up periods was often not present or was ambiguous, raising questions as to long-term impact; only Mahoney *et al.* (2003) and Eisdorfer *et al.* (2003) described follow-up periods that extended beyond the end of the intervention.

### Measures

Most studies used multiple outcome measures, which is important in order to capture the range of possible impacts on carers. In several, however, multiple measures were used but only certain outcomes were reported, and in some cases, only certain components of measures used were analyzed (e.g., Marziali and Donahue, 2006; Finkel *et al.*, 2007). It was also not always made explicit why certain measures were chosen. There is a question in the wider literature as to what constitutes effectiveness, and Magnusson *et al.* (2005) call for meaningful, realistic outcome measures with a clearer conceptual link with the intervention aims. In some studies, this link was somewhat tenuous. For example, Rosen *et al.*'s (2003) intervention aimed to increase family participation in nursing home care, but assessed carers’ knowledge of dementia.

### Outcome domains

Studies used a wide range of carer self-report measures to evaluate interventions, and most used more than one outcome measure. The review of findings of studies is grouped according to outcomes measured, and consequently the same study may be reviewed in more than one section.

#### Mood and mental health

Eight studies used measures of carer mental health, including measures of depression, anxiety, and general mental health.

##### Depression

Seven studies measured depression. All used variants of the Center for Epidemiologic Studies Depression Scale (CES-D; Radloff, 1977). Four high quality studies found either an improvement in CES-D score (Eisdorfer *et al.*, 2003; Beauchamp *et al.* 2005), or partial effects on depression, in terms of finding an effect on a subscale, or among a subgroup of participants (Mahoney *et al.*, 2003; Gallagher-Thompson *et al.*, 2010). Three medium quality studies found either no main effect on depression (although post-hoc effects) (Finkel *et al.*, 2007), no effect on depression (Marziali and Donahue, 2006), or did not report the results despite measuring depression (Marziali and Garcia, 2011).

Beauchamp *et al.* (2005) found a dose–response relationship showing that greater time spent viewing the internet program was associated with greater improvements on a composite outcome measure, which included depression. There are some tentative suggestions that other factors may moderate the relationship between intervention and depression. Eisdorfer *et al.* (2003) found that the efficacy of the intervention differed according to ethnicity and type of carer, in terms of their relationship to the person being cared for, and Finkel *et al.*'s (2007) post-hoc tests found that carers with greater baseline depression demonstrated greater improvements in depression.

##### Anxiety

Two high quality studies (Mahoney *et al.*, 2003; Beauchamp *et al.*, 2005) measured state anxiety using the State-Trait Anxiety Inventory (STAI; Spielberger *et al.*, 1985). Evidence from these two studies suggests that the computer-mediated interventions reduced carer anxiety, although Mahoney *et al.* (2003) only found this result in relation to participants with medium or low levels of mastery at baseline.

##### General mental health

One study of Marziali and Garcia (2011) measured general mental health using the mental health subscale of the Health Status Questionnaire 12 (HSQ-12, Pettit *et al.*, 2001). Significant results were only obtained after conducting multiple post-hoc tests without correction for family-wise error rate. Torp *et al.* (2008) used the General Health Questionnaire 20 (Goldberg and Williams, 1991) and did not find an effect. Beauchamp *et al.* (2005) used two subscales from the Revised Ways of Coping (Vitaliano *et al.*, 1985) in their higher quality study, but did not find any differences over time between the control group and the treatment group.

#### Carer physical health and health Behaviors

Marziali and Donahue (2006) measured carer physical health using the Health Status Questionnaire 12 and Finkel *et al.* (2007) measured carer self-care activities using a modified version of the Caregiver Health and Health Behaviors Scale (Posner *et al.*, 1993). Neither study reported any effects.

#### Carer stress and burden

Nine studies measured carer stress or burden. Four used variants of the Revised Memory and Behaviour Problems Checklist (RMBPC, Teri *et al.*, 1992) (Mahoney *et al.*, 2003; Marziali and Donahue, 2006; Finkel *et al.*, 2007; Gallagher-Thompson *et al.*, 2010) and the other studies used a variety of other measures. Five studies of medium and high quality found positive effects of the intervention on carer burden (Glueckauf *et al.*, 2004; Beauchamp *et al.*, 2005; Gallagher-Thompson *et al.*, 2010; van der Roest *et al.*, 2010; Marziali and Garcia, 2011). Three studies of medium and high quality found some effects of the intervention on carer burden (Mahoney *et al.*, 2003; Marziali and Donahue, 2006; Chiu *et al.*, 2009). Mahoney *et al.* (2003) found that participants with lower mastery at baseline showed greater improvements in the bothersome nature of caring, and that wives showed a decrease in the bothersome nature of caring compared to husband, child, sibling, or other relation carers. Marziali and Donahue (2006) found that the intervention group experienced a reduction in stress. There is some limited evidence of dose–response relationship: Chiu *et al.* (2009) found that change on the Burden Scale for Family Caregivers (BSFC, Gräsel *et al.*, 2003) was not significant overall, but that frequent users had greater change than nonusers. One study found no effect on carer burden as a result of the intervention (Torp *et al.*, 2008).

In a high quality study, Gallagher-Thompson *et al.* (2010) found that mean upset or bother in relation to problems decreased in a skills learning DVD group, but not in an educational DVD group, perhaps indicating that information needs to be combined with other forms of support to reduce carer burden.

#### Carer social support

Three medium quality studies used measures of social support (Finkel *et al.*, 2007 – A received social support scale from the Inventory of Socially Supportive Behaviours, Barrera *et al.*, 1981; Marziali and Donahue, 2006 – The Multidimensional Scale of Perceived Social Support, Zimit *et al.*, 1988; Torp *et al.*, 2008 – the Revised UCLA Loneliness Scale, Russel *et al.*, 1980, and the Family and Friendship Contacts Scale, Andersson, 1984) with varied findings.

Finkel *et al.* (2007) found that although there were no main effects for received social support, those in the intervention condition with higher levels of support at baseline were more likely to maintain that support than those with lower levels of support. Marziali and Donahue (2006) did not find a difference between intervention and control groups in relation to perceived social support at follow-up. Torp *et al.* (2008) found increases in social contacts and social support at follow-up. This is perhaps not a surprising finding given that a significant part of the intervention involved introducing computer novices to the internet, which would be likely to increase their capacity for social networking.

#### Positive aspects of caregiving

Two studies used measures of the positive elements of caregiving (Beauchamp *et al.*, 2005 – Positive Aspects of Caregiving, Tarlow *et al.*, 2004); Glueckauf *et al.*, 2004 – Stress-Related Growth Scale, Parke *et al.*, 1996). The high quality study (Beauchamp *et al.*, 2005) found that the intervention increased positive elements of caring, but the medium quality study (Glueckauf *et al.*, 2004) did not.

#### Carer self-efficacy

One medium quality study (Glueckauf *et al.*, 2004), found that carers reported pre to post improvements on all three subscales of the Caregiving Self-Efficacy scale (Steffen *et al.*, 2002). Another medium quality study (van der Roest *et al.*, 2010) did not find an effect of the intervention on carer self-efficacy. A high-quality study (Beauchamp *et al.*, 2005) asked participants six “self-efficacy questions” and found that compared to the control group, the treatment group reported greater gains with respect to self-efficacy.

#### Program impact measures and composite measures

Five studies used items or measures that asked participants how the intervention had changed aspects of caring for them (Magnusson *et al.*, 2005; Torp *et al.*, 2008; Gallagher-Thompson *et al.*, 2010; Lewis *et al.*, 2010; van der Roest *et al.*, 2010). In this review, these measures are referred to as program impact measures. One additional study used a 16-item knowledge questionnaire (Rosen *et al.*, 2003). All but one of these studies found positive effects on these measures. For the most part, the studies that used these measures were of poorer quality. This is, in part, because studies that use such measures as their only outcome measure cannot score highly on the modified version of Downs and Black's (1998) quality assessment tool.

Lewis *et al.* (2010) was one of two studies that only used a program impact measure. Participants generally endorsed finding the intervention to be educational, convenient, useful, and interesting. Lewis *et al.* (2010) generally did not find any significant relationships between demographic data and responses, apart from a low correlation between age and the caregiving scale; as age increased, scores on the caregiving scale decreased, indicating less perceived benefit from the intervention.

In Torp *et al.*'s (2008) study, at follow-up, carers reported less need for information about the cared-for person's illness and caring. However, whether this knowledge would have equated to an increase in ability or confidence in caring for the person cannot be established.

Rosen *et al.* (2003) used a 16-item knowledge questionnaire before and after a computer-based education intervention for family members of PwD who were nursing home residents. They found that knowledge of key principles of dementia care improved after the intervention. It is worth noting, however, that the mean score on the knowledge questionnaire increased by 2.3 items. While this was statistically significant, such an increase may not mean much in practice.

Van der Roest *et al.* (2010) found no difference between the control group and the intervention group's knowledge about services following the intervention. They also found that the intervention group's mean opinion on the usefulness of the intervention was neutral.

## Discussion

Sixteen papers describing 14 studies met inclusion criteria. Interventions were varied and multifaceted, with a range of different outcome measures. These factors make direct comparison of studies difficult, in line with previous reviews of support for carers of PwD (e.g., Wu *et al.*, 2009). All studies found some positive effects of the intervention evaluated, although the lack of statistical power of many studies and the use of some nonstandardized measures further complicates comparison of studies.

Having said this, the most commonly measured variables were carer burden/stress and depression. In general, higher quality studies found that interventions did have an effect on these variables. Two high quality studies also found that anxiety was reduced following intervention. Positive aspects of caring may also be increased through these interventions as may through carer self-efficacy. There were mixed results in relation to social support, and physical aspects of caring did not seem to be affected. Program impact measures indicate general acceptability of these interventions.

### Factors influencing the effectiveness of interventions

Due to interventions being multicomponent and complex, it is difficult to disentangle the efficacy of individual intervention components, with a large range of factors having potential effects, including the intensity and duration of the intervention, carer characteristics, and outcome selected (Cooke *et al.*, 2001). Several of the reviewed studies attempted to examine factors influencing outcomes, although this was rarely the main focus and there was no obvious overarching framework guiding which factors to examine. Two studies found that intervention effects were moderated by certain baseline characteristics of participants, with the intervention having greater effect for those with lower mastery (Mahoney *et al.*, 2003) or greater depression (Finkel *et al.*, 2007). An interesting speculation is that this could indicate that the interventions are more effective for those who have more problems to start with. It could, however, also simply represent regression to the mean. As found in earlier research (e.g., George and Gwyther, 1986), two high quality studies (Eisdorfer *et al.*, 2003; Mahoney *et al.*, 2003) found differential effects of interventions according carer–care recipient relationship and ethnicity. These findings caution against generalizing results of research too widely and highlight the need for such factors to be considered in the design of interventions for carers of PwD, something which is perhaps more easily incorporated in multimedia than traditionally delivered interventions. It is also potentially the case that educational information alone does not have much effect and that it needs to be combined with learning skills (Gallagher-Thompson *et al.*, 2010). One possible issue with computer-mediated interventions is the need for carers to be self-motivated; one high quality study (Beauchamp *et al.*, 2005) highlighted this when they found a dose–response relationship between viewing the intervention program and change in a global composite outcome variable.

### Methodological issues

The studies reviewed had a number of limitations: they often had poorly defined aims, many did not have control groups, a number were underpowered and follow-up periods were often short. Future research would benefit from addressing these issues. It is also important that outcome measures be closely linked to the aims of interventions in order that the intervention's effectiveness can be properly evaluated.

### Clinical implications and future research

Computer-mediated intervention for informal carers of PwD is a growing area; these interventions offer a range of potential benefits. This review found that their effectiveness is mixed, but generally positive. This suggests that it would be beneficial to carers, and also to services—in terms of reaching more carers as well as potential cost saving implications—for this medium of intervention to be developed so that more individuals can benefit. As home computer use and mobile devices become increasingly widespread, the viability of computer-mediated interventions increases, and the cost to service providers decreases since increasing numbers of carers already have the requisite hardware. More research is, however, needed in order to ensure that interventions are maximally effective. Research needs to consider the effects of interventions on people of different ethnicities and carer–care recipient relationships, as there is evidence that differential effects exist between groups.

Although it was beyond the scope of this review to consider the qualitative components of studies, information gathered from interviews with carers who have used computer-mediated interventions offers an important supplement to quantitative outcome measures. It provides deeper insight into carers’ experiences of using the interventions, and is able to go beyond measurement of symptom reduction and tap into factors, such as feeling supported and less alone, that are of great importance to carers. Future research into computer-mediated interventions for carers of PwD would benefit from a mixed-method approach.

## Conflict of interest

None

## Description of authors’ roles

Vicky McKechnie conducted this literature review as part of her DClinPsy thesis. She located and evaluated the papers in this review, and wrote the first draft of the paper.

Joshua Stott was the joint supervisor for Vicky's DClinPsy thesis. He provided supervision throughout the project and he has commented on and edited several drafts of the paper.

Chris Barker was the joint supervisor for Vicky's DClinPsy thesis. He provided supervision throughout the project and he has commented on and edited several drafts of the paper.
